# Syngenta's contribution to herbicide resistance research and management

**DOI:** 10.1002/ps.6072

**Published:** 2020-09-21

**Authors:** Shiv Shankhar Kaundun

**Affiliations:** ^1^ Herbicide Bioscience, Syngenta Jealott's Hill International Research Centre Berkshire UK

**Keywords:** Syngenta, herbicide resistance research, detection methods, simulation modelling, integrated weed management

## Abstract

The evolution of weed resistance to herbicides is an ever‐increasing problem that affects crop yield and food production. In Syngenta, we believe that this difficult and complex issue can be most efficiently addressed through a deep understanding of the evolutionary dynamics and mechanism of resistance. A profound knowledge of resistance is key to developing the next generation of resistance‐breaking compounds with existing or novel herbicide sites of action. We use a multidisciplinary laboratory‐based, glasshouse and field biology approach to study herbicide resistance and provide strong science‐based solutions to delay the onset and manage resistance. We have developed and implemented simple early‐season resistance detection methods to allow farmers make an informed decision for effective weed control. We have built mechanistic, individual‐based computer models to design profitable, long‐term sustainable weed management programs. Our zero tolerance approaches employ herbicides with different sites of action, applied in mixtures and sequences, to minimise the risk of resistance evolution. Weeds are targeted at the right growth stage with optimal herbicide formulation and spray technology for maximising weed control and depleting the seed bank. We are promoting the use of competitive crop varieties and other nonchemical methods for an integrated weed management strategy. We have a global web of external collaborations for studying and managing herbicide resistance. We are committed to farmers' education and training on herbicide resistance, and regularly share our methods and findings via conferences and peer‐reviewed scientific publications for the benefit of the wider weed science community and field practitioners. © 2020 Society of Chemical Industry

## Graphical abstract

Overview of Syngenta's activities for the advancement of herbicide resistance science and management.

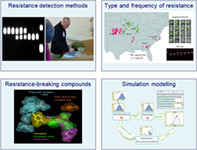

## 1 INTRODUCTION

Farmers need to protect their fields from weeds, insects and diseases in order to grow a crop profitably. Weeds are particularly damaging pests that compete with crops for space, light, water and nutrients.^1^ They are highly adaptative foes that can cause an average 35% yield loss if not properly controlled in major crops worldwide.^2^ Weeds have been effectively managed using synthetic herbicides since their discovery more than 70 years ago.^3^ However, their extensive use over time, often as the sole control method, has resulted in the evolution of weed resistance in all major agro‐systems.^4^ Most herbicides are concerned, including compounds that were originally thought to be characterised by a low risk of resistance evolution either because they are poorly metabolisable by plants or they target multiple and difficult to mutate enzymes.^5^, ^6^, ^7^, ^8^ To date, herbicide resistance has been documented in 152 broadleaf and 110 grass species from 70 different crops.^9^ The most problematic weeds are *Lolium rigidum* from Australia, *Alopecurus myosuroides* from north‐western Europe and *Amaranthus palmeri/tuberculatus* from corn‐soybean production areas in the USA, to name but a few.^10^ These highly polymorphic weed species are often resistant to multiple herbicide sites of action, leaving few options for effective chemical weed control.^4^ The yield loss due to herbicide‐resistant *A. myosuroides* in wheat in the UK is estimated at 0.8 million tons (equivalent to £400 million loss in profit) per year, while the increase in cost due to glyphosate resistance in the USA is in excess of several billion dollars annually.^11^, ^12^

Resistance can be endowed by a target‐site modification/overexpression, enhanced metabolism, sequestration and reduced uptake/transport of the herbicides.^13^ Several studies have shown that resistance often evolves from the high genetic standing variation within weed populations.^14^, ^15^ Resistance can also be transferred to neighbouring fields by pollen or seed movement.^16^ Long‐distance transmission via seed or machinery contamination is not uncommon, as exemplified by glyphosate‐resistant *A. palmeri* introduced in Argentina via contaminated crop seed import from the native USA.^17^, ^18^ Weed resistance can be acquired by cross‐species hybridisation or introgression from phylogenetically close herbicide‐resistant crops.^19^ Resistance is also favoured by the application of suboptimal herbicide rates allowing individual weeds to accumulate minor genes which would otherwise be killed by the toxophore.^20^, ^21^ The expression of herbicide resistance can be plant growth stage and temperature dependent, and controlled by single or multiple genes.^22^, ^23^, ^24^ Resistant plants can be characterised by pleiotropic effects or can be as fit as susceptible individuals.^25^ Resistance to one herbicide can cause weed control failures to compounds belonging to different herbicide sites of action via a nontarget‐site based mechanism.^26^, ^27^, ^28^

No new herbicide mode of action has been commercialised over the last 30 years and current herbicides face stricter regulatory hurdles upon re‐registration, putting enormous pressure on existing compounds.^29^ There is growing practical evidence of the difficulty of chemically controlling weeds that have been primarily managed by herbicides in the past.^30^ Herbicide resistance is clearly a multifaceted problem that is affecting the livelihoods of farmers and threatening food security.^10^, ^31^ Tackling herbicide resistance is therefore of prime importance for a major agrichemical company such as Syngenta. In this paper, we provide an overview of Syngenta's activities for the advancement of resistance research and management.

## 2 DEVELOPMENT OF HERBICIDES FOR THE CONTROL OF SENSITIVE AND RESISTANT WEED POPULATIONS

Over the years, Syngenta and its legacy companies have developed several vital herbicides that have greatly benefitted agriculture. These include paraquat, atrazine, mesotrione, fomesafen and *S*‐metolachlor, which inhibit photosystem I, photosystem II, 4‐hydroxyphenylpyruvate dioxygenase, protoporphyrinogen‐oxidase and very long chain fatty acid synthesis, respectively. Historically, potency, weed spectrum, crop selectivity, safety to operators and the environment, as well as the cost to produce the herbicide have been the major factors taken into consideration. Nowadays, the ability to control resistant weeds has become as important, especially when the target weeds can no longer be killed with available herbicides. A novel site‐of‐action herbicide will control target‐site resistance to all current herbicides, but it may still be affected by the generalist nature of nontarget‐site resistance mechanisms.^27^, ^28^ On the other hand, compounds from known sites‐of‐action can sometimes be effective against resistance selected by earlier chemotypes due to differential binding, metabolism and transport of individual herbicides.^32^ At Syngenta, both known and novel herbicide sites‐of‐action are explored for controlling resistant weeds.

Most of the weed populations that have been extensively studied over the years habitually display phenomenal resistance to multiple herbicide sites‐of‐action.^33^, ^34^, ^35^ Whilst interesting from an academic standpoint, these populations are often of little practical importance because they are not representative of the vast number of sensitive and resistant populations from farmers' fields. To develop a successful resistance‐breaking herbicide, a thorough understanding of the type and frequency of the prevailing resistance mechanisms is essential. It is also important to foresee any future resistance mechanisms to account for the 10 or more years' lag between herbicides in research and development and commercial launch. This is achieved by careful analysis of current and future herbicide use patterns and the weeds' propensity and manner to evolve resistance. To gain an understanding of resistance in the target weeds, large numbers of seed populations are regularly sampled from relevant field control failures for further characterisation in our glasshouses and laboratories. On resistance confirmation using whole‐plant tests, target‐site resistance is assessed with established Sanger sequencing and biochemical assays. quantitative Polymerase Chain Reaction (QPCR) and Enzyme‐Linked Immunosorbent Assay (ELISA) methods are also used for determining whether resistance is due to an overexpressed target enzyme. Nontarget‐site resistance is investigated using a range of radiolabelled and cold‐herbicide techniques, including high‐throughput liquid chromatography coupled with mass spectrometry. An *in silico* genomic analysis and an association mapping and transcriptomic approach are increasingly being employed to identify the most common nontarget‐site resistance genes present in the target weeds. The pre‐selection of novel compounds is often aided by computational and crystallography methods to avoid the most prevalent resistant mechanisms identified in the weed populations. Individual resistance genes are heterologously expressed in model organisms and assayed to allow for cost‐effective structure–activity relationship studies. Promising candidates are further tested on a set of representative weed samples under glasshouse conditions before being assessed on known resistant populations in the field. The design–synthesis–test–learn cycle is repeated until a valid resistance‐breaking compound is identified for development and commercialisation.

Pinoxaden, developed by Syngenta for the selective control of key grass weeds in small grain cereal crops, is a compound that has a competitive edge over other previously marketed cereal‐safe acetyl‐CoA carboxylase (ACCase) inhibiting herbicides.^36^ The compound is structurally different from aryloxyphenoxypropionates (FOPs) and cyclohexanediones (DIMs) to merit classification into a different phenylpyrazolin (DEN) ACCase‐herbicide subgroup.^37^ Importantly, pinoxaden binds to a slightly different region of the ACCase carboxyl transferase domain compared to FOP herbicides.^38^ Consequently, it is less affected by resistance endowed by some FOP‐specific resistant mutations in several key grassweeds.^39^ Pinoxaden was also found not to be significantly impacted by metabolism selected by previous cereal‐selective ACCase grassweed killers in most *Lolium* spp. and *Avena* spp. populations, for example.^40^, ^41^, ^42^ Although pinoxaden is affected by some FOP‐DIM‐DEN target‐site mutations, it remains the leading ACCase‐inhibiting herbicide of choice for the post‐emergence control of grass weeds in wheat, barley and triticale due to its favourable resistance profile.^43^

## 3 DEVELOPMENT AND IMPLEMENTATION OF EARLY‐SEASON HERBICIDE RESISTANCE DETECTION METHODS

Several methods have been developed for confirming evolved weed resistance to herbicides.^44^ These include seed or pollen‐based Petri dish as well as a few sophisticated physiological, biochemical, molecular and hyperspectral assays.^45^, ^46^, ^47^ In most instances, however, resistance is still determined by the use of the classical whole‐plant pot assays with seeds collected from field survivors and tested under controlled glasshouse conditions alongside a standard sensitive population.^48^ Whilst reliable and most representative of field situations, the whole‐plant pot method is a laborious, space‐inefficient and time‐consuming procedure that can take up to 8 weeks to complete. As the test requires seeds, it does not permit an educated choice of herbicides for effective weed control at the beginning of the current growing season.

In Syngenta, we have used our deep knowledge of ACCase herbicides to develop molecular assays for detecting resistance before field application. For example, our initial large‐scale screening of *Avena fatua* populations from Western Canada has shown that pinoxaden is mainly affected by the three I1781L, D2078G and C2088R FOP‐DIM‐DEN ACCase mutations but not by FOP‐specific target‐site and metabolic resistance. Consequently, we have developed and locally implemented three DNA‐based derived cleaved amplified polymorphic sequence (dCAPS) assays for identifying these three mutations, using a piece of pretreated *A. fatua* leaf as starting material.^49^ We confidently recommended the application of pinoxaden to advisors and farmers for successfully controlling *A. fatua* in the absence of the target‐site resistance mutations. The results could be generated within 24–48 h after the leaf fragments were received at a central molecular biology laboratory established in Western Canada. Although effective, the DNA‐based methods required qualified personnel to run, and the results and advice were only valid for this specific herbicide, weed species and wheat‐growing region.

Subsequently, a more widely applicable, early‐season assay, denoted the Syngenta Resistance In‐Season Quick (RISQ) test, was developed for detecting resistance in a range of grass and broadleaf weeds.^50^, ^51^ The Syngenta RISQ test involves seedlings collected from farmers' fields prior to herbicide application. The seedlings are sent to a designated testing centre and transplanted onto agar containing informative rates of herbicides. Survivorship is recorded 7–14 days later and compared to known standard sensitive and resistant populations. The method can be applied to all herbicides irrespective of the mechanism of resistance. The Syngenta RISQ test does not require a glasshouse and can be conducted in a basic, ventilated room using natural or artificial light. The method was successfully implemented in a number of countries, including India, for the detection of resistance to ACCase and acetolactate synthase (ALS)‐inhibiting herbicides in *Phalaris minor* from wheat fields.^52^ Fifteen testing centres were established in the highly affected Indian states of Haryana and Punjab, and tests were carried out on samples collected from more than 4500 fields during 2017–2019. The results were fed back to the farmers in good time at the start of the growing season to allow for an optimal herbicide programme to effectively control *P. minor*. The method is currently being used in Pakistan for large‐scale testing of *P. minor* and is also available in New Zealand for identifying resistance in *L. rigidum*.

The published Syngenta RISQ test has been adopted by several other independent weed science practitioners. For instance, researchers at the Institut National Agronomique de Tunisie (INAT, Tunisia) have cost‐effectively employed the method to map resistance to ALS and ACCase herbicides in *Lolium multiflorum* from Northern Tunisia.^53^ The Syngenta RISQ test was also used by the Agricultural Development and Advisory Service (ADAS), the largest UK‐based independent agricultural and environmental consultancy provider, for surveying potential glyphosate resistance evolution in the highly damaging *A. myosuroides* species.^54^ Glyphosate is a very important preplant burndown and chemical fallow herbicide for managing black‐grass in the UK. Black‐grass resistance to glyphosate is not yet documented in the country but there is growing evidence of difficulty in controlling black‐grass populations in areas where the herbicide has been used extensively.^55^ As the Syngenta RISQ test is capable of identifying resistance before seed production, it is proactively being employed by ADAS to detect the potential onset and spread of glyphosate‐resistant *A. myosuroides* in the UK.^54^

## 4 SIMULATION MODELS FOR PREDICTING THE EVOLUTION OF HERBICIDE RESISTANCE

The evolution of weed resistance to herbicides is complex and determined by many interacting biological, genetic and agro‐ecological parameters.^56^ These include the weed mating system, seed bank characteristics, the initial frequency, dominance and potential fitness cost associated with the resistant trait, as well as the crop rotation, tillage and herbicide program.^57^ In some instances, several hundred weed control scenarios could be envisaged based on the crop rotation pattern, conventional and increasingly stacked genetically‐modified crop varieties, use of herbicides solo or in two‐, three‐ or even four‐way mixtures, timing of application and formulation type, which make these many scenarios difficult to assess using classical field or laboratory experiments. Computer‐based simulation models have proved useful to decipher how these diverse parameters interact to modulate the evolutionary trajectory and population dynamics of herbicide resistance.^58^ Whilst the models have grown in complexity over time, most of them assume a single gene effect and represent an annual weed.^59^ However, there is growing evidence that herbicide resistance is mainly caused by polygenic nontarget‐site based mechanisms and a significant number of problematic weed species display a perennial life cycle.^9^, ^27^, ^60^

In Syngenta, we have built a generalised individual‐based model (IBM) to account for both single and multigene quantitative resistance effects in annual weeds.^61^ The IBM incorporates the whole life history of the weed and analyses how different traits and management scenarios impact the evolution of resistance in the species. Two main outputs are generated, namely, the density of surviving weeds and the corresponding risk of herbicide resistance evolution. Application of the model to *A. tuberculatus* from corn‐soybean agro‐systems in the Midwestern USA revealed the significance of the weed's genetics and management practices as compared to seed germination and mortality to the evolution of glyphosate and mesotrione resistance in the species.^61^ The high phenotypic variation, prolific seed production and pre‐existing level of resistance alleles were particularly impactful. The annual model was also used to investigate the sustainability and economics of early weed management with pre‐emergent residual compounds and stacked herbicide‐tolerant traits offering additional chemical weed‐control options.^62^ Simulation results showed that both approaches were effective at controlling *A. tuberculatus*. The post‐emergence programs needed additional chemical and nonchemical tactics to maintain the benefits provided by the stacked herbicide resistant traits, whereas the pre‐emergence components necessitated at least two effective herbicides belonging to two different sites of action. Whilst requiring slightly higher investment, the proactive weed control approach was significantly more profitable in the long term as opposed to a less diversified herbicide program.^62^

We have also constructed a second generalised IBM to represent the life cycle of perennial weed species characterised by both sexual and asexual reproductive systems.^63^ The model was applied to investigate growing resistance to glyphosate (quantitative trait) and ACCase‐inhibiting herbicides (single‐gene mutation) in *Sorghum halepense* from soybean production systems in Argentina. Simulation results indicate that resistance endowed by single‐gene mutations was mainly influenced by the initial level of resistant alleles and the associated pleotropic effects. The population dynamics of *S. halepense* was driven by evolved resistance, soil tillage and rhizome fecundity. The model suggested that herbicides should be combined with some level of soil tillage to address the limited chemical options for rhizome control to maintain long‐term effective management of *S. halepense*.

Our generalised annual and perennial models are flexible to allow resistance prediction for a wide range of weed species, as long as the corresponding parameters and management options are incorporated. The models are currently being used to design sustainable weed control strategies for a number of problematic species, such as *P. minor* in India, *Raphanus raphanistrum* in Australia, *A. myosuroides* in the UK, *A. palmeri*/*A. quitensis* in Argentina and *Digitaria insularis* in Brazil. The simulation models are also being employed in assessing the impact of the potential loss of glyphosate in some major European countries and the effect of regulatory‐driven herbicide rate reduction on the evolution of resistance in key weed species. Additionally, the models are utilised for educational purposes and for demonstrating the value of an integrated weed management (IWM) strategy combining chemical and nonchemical methods for effective weed control.

## 5 DEVELOPMENT OF SUSTAINABLE WEED MANAGEMENT PROGRAMS

Weeds have continually adapted to human‐directed intervention since the dawn of agricultural production. This is demonstrated by the evolution of resistance due to over‐reliance of herbicides as the primary method of weed control in modern agriculture.^4^ Model simulations and field observations suggest that the risk of resistance can be mitigated by alternating and mixing herbicides with different sites of action.^64^, ^65^ The strategy is more effective if the herbicides are used at full labelled rates prior to resistance evolution to one component of the mixture.^66^ Accordingly, we have developed increasingly diverse chemical programs with highly optimised herbicide formulations to achieve maximum kill and deplete the weed seed bank. The double‐knock strategy, for instance, consists of applying a second product to control weeds that have escaped a first herbicide application.^67^, ^68^ One such double‐knock approach endorsed by Syngenta is a preplant burndown application of glyphosate + clethodim followed by paraquat + *S*‐metolachlor 14–21 days later to control increasingly problematic resistant *D. insularis* populations in soybean in Brazil. A second concept to delay the onset of resistance is to control weeds early with a mixture of pre‐emergence herbicides to relieve the pressure on subsequent post‐emergence applications. Early weed management (EWM) protects yield by targeting weeds when they are most vulnerable and less inclined to express some metabolic and even certain target‐site resistant mechanisms.^69^, ^70^, ^71^ Syngenta recommends a mixture of prosulfocarb [Herbicide Resistance Action Committee (HRAC) group N], flufenacet (HRAC group K3) and diflufenican (HRAC group F1) for the pre‐emergence control of black‐grass in wheat in Europe. The pre‐emergence treatment can be complemented by a foliar application of pinoxaden (HRAC group A) + pyroxsulam (HRAC group B) in spring to complete the level of grass weed control. The use of five different sites of action is more sustainable than a single application of a post‐emergent ACCase or ALS‐inhibiting herbicide as commonly practiced until recently. Syngenta has also developed a blend of mesotrione (HRAC group F2), bicyclopyrone (HRAC group F2), atrazine (HRAC group C1) and *S*‐metolachlor (HRAC group K3) for the control of over 70 different grass and broad leaf weeds in corn.^72^ The four‐way mixture has a flexible window of application spanning from 28 days preplant to 12‐in. corn stage, and provides up to 50 days residual control of weeds including the increasingly troublesome *A. palmeri* and *A. tuberculatus*. The post‐emergence application of a three‐way mixture of glyphosate (HRAC group G), mesotrione and *S*‐metolachlor in addition to atrazine within 21 days of the pre‐emergence application is advocated for season‐long, overlapping residuality in glyphosate‐tolerant corn. The synergistic effect of atrazine and mesotrione in the four‐way mixture and other related products further protects against resistance evolution in key weeds.^73^, ^74^ Similar season‐long residual programs include a mixture of metribuzin (HRAC group C1) and *S*‐metolachlor applied pre‐emergence followed by *S*‐metolachlor + fomesafen (HRAC group E) 30 days later for controlling glyphosate‐resistant *A. palmeri* and *A. quitensis* populations in soybean in Argentina. The use of three different sites of action was shown to have a more favourable weed control and lower resistance evolution risk than repeated applications of protoporphyrinogen oxidase (PPO)‐inhibiting herbicides within the soybean growing season.^75^ In rice, Syngenta recommends the pre‐emergence use of pretilachlor (HRAC group K3), a multisite herbicide that inhibits very long chain fatty acid synthesis, for decreasing the selection pressure on ACCase and ALS‐inhibiting herbicides applied post‐emergence for the control of important grass weeds such as *Echinochloa crus‐galli* and *Leptochloa chinensis* in South‐East Asia.

**Figure 1:**
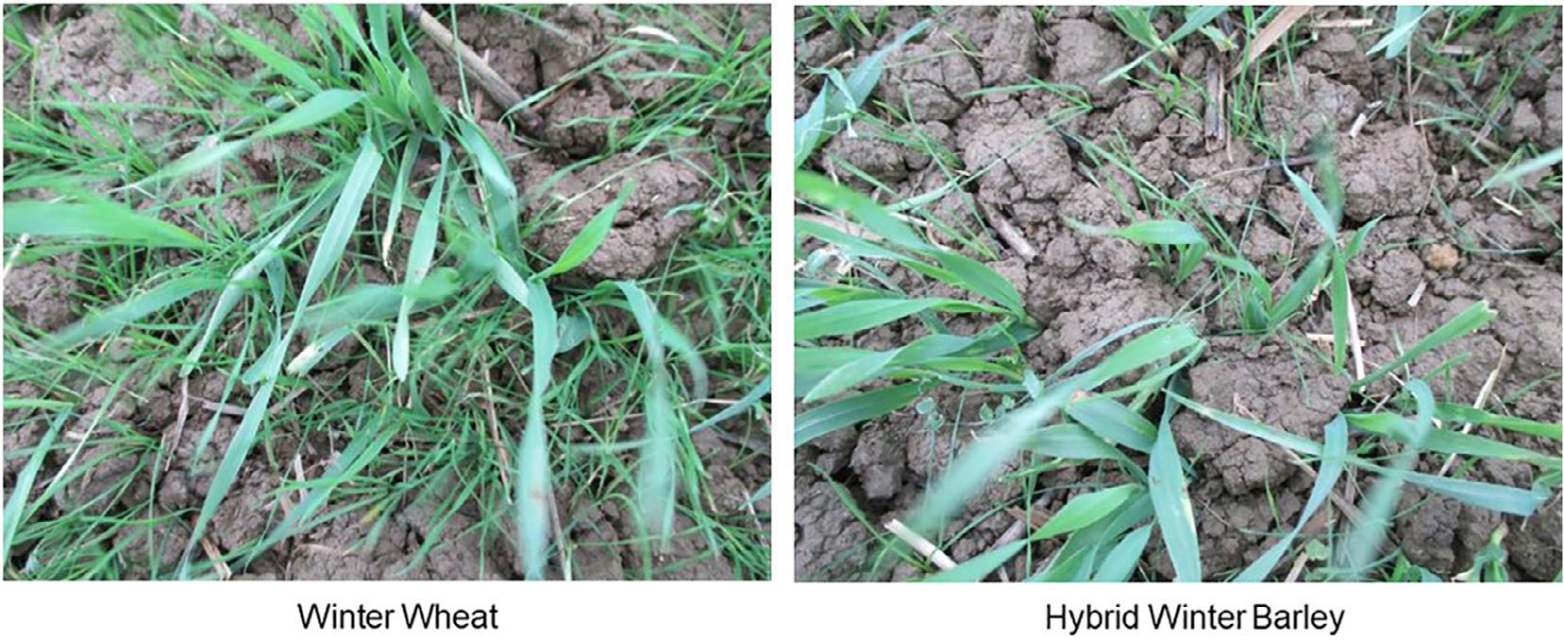
Reduction of black‐grass seed return in Syngenta's hybrid winter barley compared to winter wheat.

To further extend the longevity of herbicide programs, Syngenta is promoting an IWM strategy that combines several cultural, mechanical, biological and chemical weed control methods.^76^ Nonchemical methods require testing on a more case‐by‐case basis as they do not always provide consistent levels of weed control as chemical methods. Collection and destruction of seeds at crop harvest, for instance, was shown to be very effective at depleting the seed bank of *L. rigidum* in Australia, but is less applicable to species that shed most of their seeds before harvest.^77^, ^78^ Similarly, cultivation was found to be efficient at burying black‐grass seeds and limiting germination in the first year, but if repeated in the following year this method can return the buried seeds to the surface, leading to increased black‐grass germination.^79^ Syngenta has targeted different cropping systems and regions to investigate the effectiveness of nonchemical control methods to sustain our herbicidal solutions. These include the introduction of a summer crop to break the soybean monoculture prevalent in Argentina. Several different cover crops are also being considered to better manage the rising problem of glyphosate‐resistant *A. palmeri* and *A. quitensis* in the country. In France, we are promoting different nonchemical weed management strategies such as crop rotation, increased seeding rate, competitive varieties and inter‐row mechanical weed management for the control of grass weeds in wheat, barley, oil seed rape, sunflower and corn. In Northern India, we are evaluating different types of crop rotation, cultivation, seed capture and crop establishment methods to better manage multiple herbicide‐resistant *P. minor* populations in wheat. Our most advanced IWM trial for black‐grass resistance control in small grain cereal crops in the UK is a long‐term, multifactorial study being conducted at one of our innovation centres in Barton (Cambridgeshire, UK). Several important parameters are being considered, including cultivation, crop rotation, spring cropping, competitive varieties, seeding rate, water volume, boom height and nozzle type. The trial investigating three modes of cultivation, namely, 15 cm noninversion tillage, direct drill and ploughing, showed that over a 3‐year period direct drill–plough–direct drill gave on average the best level of black‐grass control whilst ensuring good crop profitability. An autumn cultivation led to a better spring crop establishment and reduction of black‐grass plants. In a scenario of early crop drilling, prominent weed density and high seed dormancy, sequencing of herbicides provided better control compared to a pre‐emergence stack of herbicides. Herbicide performance was negatively impacted by crop residues with direct drilling. Increasing seeding rates of both spring wheat and spring barley resulted in the progressive reduction of black‐grass ears per m^2^. In agreement with previously published studies, Syngenta's hybrid winter barley was better able to outcompete black‐grass as compared to a typical winter wheat or a winter barley variety.^80^ Black‐grass plants in Syngenta's hybrid barley plot were mostly below the crop canopy and produced fewer tillers and seeds than in the winter wheat and winter barley. Consequently, black‐grass in winter wheat and winter barley contributed more to the replenishment of the seed bank compared to hybrid barley (Fig. 1). The optimum speed for applying pre‐emergence herbicides was 10 km/h. An average 8% increase in grass weed control was observed when herbicides were sprayed at 200 L/ha as opposed to commonly employed 100 L/ha. Lowering the boom from 100 to 50 cm above the crop increased the efficacy of the pre‐emergence herbicide from 70% to 87%. The use of 90% drift reduction nozzles delivered consistently better levels of black‐grass control via a more even spray distribution compared to flat fan nozzles. The findings are regularly shared with local growers so that these can be trialled on their own farms (https://www.syngenta.co.uk/black-grass-barton-virtual-meeting-2020).

## 6 TRAINING, EXTERNAL COLLABORATIONS AND SCIENTIFIC PUBLICATIONS

Education and training are essential components of our herbicide resistance activities. As a committed member of the Herbicide Resistance Action Committee (HRAC) and Crop Life International, we contribute, alongside other agrichemical industries, to the establishment of guidelines for Best Management Practices (BMP). We promote herbicide mode of action labelling to encourage diversification in chemical weed control methods. We regularly produce videos and podcasts, and organise face‐to‐face training on herbicide resistance for advisors and farmers, often in collaboration with local HRACs and weed science societies. We have different web‐based resources and applications, such as ResistanceFighter.com (USA), Proximaïs (France), Spray Assist (UK) and No Malezas (Argentina) to help farmers develop an effective weed control program. We frequently organise demonstration trials for external stakeholders to emphasise the importance of recommended herbicide use rates, plant growth stages, spray technology, use of multiple effective sites of action, crop rotation and competitive varieties to reduce the risk of weed escapes and resistance onset. We have delivered hands‐on training on the Syngenta RISQ test to more than 100 external weed practitioners to drive local implementation and use. The trainees included researchers from the Indian Institute of Wheat and Barley Research (IIWBR, India), the Plant Protection Institute, (PPI, Poland), the Iran Research Institute of Plant Protection (IRIPP, Iran), the Institut National Agronomique de Tunisie (INAT, Tunisia), the Agricultural Development and Advisory Service (ADAS, UK) and the Chinese Academy of Agricultural Sciences (CAAS, China), as well as several academics from the Punjab Agricultural University (India), the University of Illinois (USA), Haryana Agricultural University (India), Prague Agricultural University (Czech Republic), Bingen University (Germany) and Nanjing Agricultural University (China), amongst others.

We have around 25 ongoing external resistance research collaborations with local and international weed scientists and herbicide resistance experts at any one time. These range from detailed mechanism of resistance studies in some increasingly problematic weed populations, to adopting a more pro‐active approach and surveying the potential onset of resistance in new species and herbicides. Typical examples include the monitoring of ALS and auxinic herbicide efficacies in broadleaf weeds in small‐grain cereal crops in Northern India and shift in ACCase herbicide activity in *D. insularis* in soybean in Brazil. These partnerships generate useful data for effectively managing the problematic weed populations with current herbicidal and nonchemical tools and for identifying compounds in research with resistance‐breaking ability. In particular, our long‐term collaboration with academics at the University of Illinois has shown that some multiple herbicide‐resistant *A. tuberculatus* populations from the Midwestern USA mimic corn to resist atrazine and mesotrione by overexpression of a single glutathione‐*S*‐transferase and by polygenic P450‐based enhanced metabolism, respectively.^81^, ^82^, ^83^ The same populations employ a rather different metabolic pathway from the crop to overcome the actions of topramezone and *S*‐metolachlor.^84^, ^85^ The multiple herbicide‐resistant populations are also able to degrade and survive some 4‐hydroxyphenylpyruvate dioxygenase (HPPD)‐inhibiting compounds that completely kill corn.^86^ A growing number of external collaborations are dedicated to generating essential weed biology and fitness data to feed into our simulation models. Seed dormancy of some problematic species is being investigated with the help of expert academics to refine our weed resistance management strategy.^87^ We are also funding studies for evaluating alternative methods such as cover crops, seed capture and inter‐row mechanical weed management to complement our chemical control options. Syngenta is one of the founding members of the International Weed Genomics Consortium (IWGC) and has teamed up with other agrichemical companies and academic researchers to create high‐quality genomes for the 20 most important weed species. Access to whole genomes will permit a more precise identification of closely related weed species and allow better tracking of the evolutionary dynamics of problematic populations. It will also facilitate the discovery of genes involved in complex nontarget‐site based resistance.

A significant proportion of our research and resistance detection methods are shared via peer‐reviewed scientific publications as well as presentations at academic conferences and farmer/advisor meetings. In the last year alone, we have contributed to several papers on simulation modelling and the mechanism of glyphosate resistance in *A. palmeri* and *A. quitensis* from soybean fields in Argentina.^62^, ^63^, ^88^, ^89^ We have published two additional papers on the novel Polymerase Chain Reaction‐Restriction Fragment Length Polymorphism (PCR‐RFLP)‐based derived polymorphic amplified cleaved sequence (dPACS) method and a corresponding dPACS 1.0 web‐based freeware (http://opendata.syngenta.agroknow.com/models/dpacs) for selecting restriction enzymes that differentiate wild‐type from mutant alleles.^90^, ^91^ The simple DNA‐based dPACS procedure offers the versatility required for genotyping highly polymorphic weeds compared to current methods which are more suited for relatively homogeneous species. The dPACS approach was illustrated by three universal assays for detecting the S264G‐*psb*A, the P106S/T/A‐*EPSPS* and the I2041N‐*ACCase* target‐site mutations that endow resistance to photosystem II, EPSPS and ACCase‐inhibiting herbicides, respectively, in a wide range of weed species.^91^ A fourth dPACS assay has helped fellow researchers discover two novel *PPX2L* mutations and nontarget‐site mechanism by confidently disregarding the commonly encountered but notoriously difficult to genotype Δ210‐*PPX2L* codon in some *A. palmeri* populations resistant to PPO‐inhibiting herbicides.^90^, ^92^, ^93^ The highly transferable Δ210‐*PPX2L* dPACS assay has been provided to academics in Argentina to allow confirmation of future cases of resistance in *A. palmeri* and *A. quitensis* that are increasingly being managed with PPO‐inhibiting herbicides. We have recently disclosed our latest method, named the Syngenta Herbicide Resistance Leaf (HRL) test, for resistance confirmation using whole detached leaves immersed in informative rates of herbicides.^94^ In line with our zero‐tolerance approach, the method was developed for identifying resistance at its very onset from a single or few field survivors, without the need and danger of letting the plants produce seeds, for subsequent characterisation in the classical whole plant pot assay. The Syngenta HRL test was successfully implemented to confirm resistance to PPO‐inhibiting herbicides in several *A tuberculatus* and *A. palmeri* populations from the Midwestern USA.^95^

## 7 CONCLUSION

Syngenta is committed to tackle the growing issue of herbicide resistance evolution in key weeds via its own research and in collaboration with local authorities, field practitioners, academics and other agrichemical industries. Our herbicide resistance research is diverse, ranging from the invention and implementation of quick resistance confirmation tests to the development and commercialisation of resistance‐breaking compounds through large‐scale resistance monitoring and mechanism studies. Our holistic approach allows the quick identification of problematic populations and the design of tailor‐made, computer‐aided sustainable weed control programs. Syngenta also continues to explore and promote nonchemical methods to complement our herbicide programs.
